# Demonstrating the Use of High-Volume Electronic Medical Claims Data to Monitor Local and Regional Influenza Activity in the US

**DOI:** 10.1371/journal.pone.0102429

**Published:** 2014-07-29

**Authors:** Cécile Viboud, Vivek Charu, Donald Olson, Sébastien Ballesteros, Julia Gog, Farid Khan, Bryan Grenfell, Lone Simonsen

**Affiliations:** 1 Fogarty International Center, National Institutes of Health, Bethesda, Maryland, United States of America; 2 School of Medecine, Johns Hopkins University, Baltimore, Maryland, United States of America; 3 New York City Department of Health and Mental Hygiene, New York, New York, United States of America; 4 Department of Evolutionary Biology, Princeton University, Princeton, New Jersey, United States of America; 5 Department of Applied Mathematics and Theoretical Physics, University of Cambridge, Cambridge, United Kingdom; 6 IMS Health, Plymouth Meeting, Pennsylvania, United States of America; 7 School of Public Health and Health Services, George Washington University, Washington, D.C., United States of America; Harvard School of Public Health, United States of America

## Abstract

**Introduction:**

Fine-grained influenza surveillance data are lacking in the US, hampering our ability to monitor disease spread at a local scale. Here we evaluate the performances of high-volume electronic medical claims data to assess local and regional influenza activity.

**Material and Methods:**

We used electronic medical claims data compiled by IMS Health in 480 US locations to create weekly regional influenza-like-illness (ILI) time series during 2003–2010. IMS Health captured 62% of US outpatient visits in 2009. We studied the performances of IMS-ILI indicators against reference influenza surveillance datasets, including CDC-ILI outpatient and laboratory-confirmed influenza data. We estimated correlation in weekly incidences, peak timing and seasonal intensity across datasets, stratified by 10 regions and four age groups (<5, 5–29, 30–59, and 60+ years). To test IMS-Health performances at the city level, we compared IMS-ILI indicators to syndromic surveillance data for New York City. We also used control data on laboratory-confirmed Respiratory Syncytial Virus (RSV) activity to test the specificity of IMS-ILI for influenza surveillance.

**Results:**

Regional IMS-ILI indicators were highly synchronous with CDC's reference influenza surveillance data (Pearson correlation coefficients rho≥0.89; range across regions, 0.80–0.97, P<0.001). Seasonal intensity estimates were weakly correlated across datasets in all age data (rho≤0.52), moderately correlated among adults (rho≥0.64) and uncorrelated among school-age children. IMS-ILI indicators were more correlated with reference influenza data than control RSV indicators (rho = 0.93 with influenza v. rho = 0.33 with RSV, P<0.05). City-level IMS-ILI indicators were highly consistent with reference syndromic data (rho≥0.86).

**Conclusion:**

Medical claims-based ILI indicators accurately capture weekly fluctuations in influenza activity in all US regions during inter-pandemic and pandemic seasons, and can be broken down by age groups and fine geographical areas. Medical claims data provide more reliable and fine-grained indicators of influenza activity than other high-volume electronic algorithms and should be used to augment existing influenza surveillance systems.

## Introduction

The last decade has seen dramatic developments in influenza surveillance systems at the regional and national scales. In the US however, despite intensified surveillance for influenza-like-illness (ILI) and laboratory-confirmed virus activity [1], the volume of information remains too sparse for detailed analyses at the state and city levels [2]. Novel electronic surveillance data streams such as Twitter and Google Flu Trends provide much higher volume information; however these algorithms do not always accurately capture local or national influenza patterns, especially during pandemics or unusual epidemics [3], [4]. Indicators based on emergency department visits provide solid localized information on a variety of influenza-related syndromes in near real-time, however, these data are not available throughout the US. In contrast to influenza, relatively little attention has been focused on respiratory syncytial virus (RSV), although the burden of this pathogen is increasingly recognized, particularly among pediatric age groups [5], [6], [7], [8]. In the absence of an RSV vaccine, it is important to optimize the timing of RSV prophylaxis in high-risk infants according to the local RSV season, requiring the need for improved RSV surveillance locally [9], [10].

Electronic medical claims data provide a unique source of information on diagnoses made by physicians and are routinely used by pharmaceutical companies to monitor disease incidence and anticipate drug or vaccine sales. So far however, this resource has remained largely untapped by epidemiologists and public health researchers. A few promising studies have suggested that electronic claims data may be useful to monitor disease patterns of diarrheal and respiratory viruses in the US and evaluate pediatric vaccine coverage in Germany [11], [12], [13], [14]. Here we demonstrate the use of electronic medical claims records to monitor local and regional respiratory virus activity during pandemic and inter-pandemic seasons in the US.

## Data and Methods

### Ethics

All patient records and information were anonymized and de-identified prior to being handed over to researchers; all records were part of routinely collected information for health insurance purposes. Dr Farid Khan, Director of Advanced Analytics, IMS Health, granted access to the patient data. The database is not accessible online but researchers interested in gaining access to the data should refer to the IMS Health website: http://www.imshealth.com/portal/site/imshealth. In keeping with similar epidemiological analyses of large-scale insurance administrative databases, no institutional board review was sought. Further, all statistical analyses were based on aggregated incidence time series rather than individual patient-level information.

### General approach

Our general approach is to compare weekly influenza indicators derived from electronic medical claims data against reference influenza surveillance time series, and against control time series unrelated to influenza (such as RSV surveillance data). Our statistical measures include correlations in weekly incidences, peak timing and seasonal estimates of epidemic intensity. Additionally we use permutation tests to show that the estimated correlations are stronger than those expected by chance between incidence time series that share common winter seasonality, so as to confirm that medical claims data capture signals truly specific of influenza activity. Analyses are conducted at the national, regional, and local scales, and stratified by age group.

### Data Sources

#### IMS Health Medical Claims Data

We used data maintained by IMS Health, a private data and analytics business that collects de-identified electronic CMS-1500 medical claim forms from full-time office-based active physicians throughout the US. Claims data are sourced from the practice management software vendors directly from the physician's office, or from the intermediary billing systems that coordinate the insurance claim transactions. In 2009, there were 560,433 active physician practices in the US of which IMS Health collected data from 354,402, or an approximate coverage rate of 61.5%. IMS Health receives the records within 1–2 weeks of the patient's visit. For validation purposes, we focused here on historic IMS Health data from July 2003 to June 2010. Claims data were kindly compiled by IMS Health for research purposes under a collaborative agreement with the authors.

Diagnoses are coded in the physician offices using international classification of diseases, 9^th^ revisions (ICD-9). We extracted visits for different outcomes, including ILI and RSV, as well as the total number of visits for any reason for denomination purposes. We created weekly time series based on the date of office visit. Several ILI case definitions were tested with the expectation that the most appropriate definition would produce a large and geographically heterogeneous spike in disease rates during the 2009 A/H1N1 influenza pandemic period, as observed in other surveillance datasets [15], and capture the timing and intensity of influenza epidemics in the pre-pandemic period. Further, a suitable ILI definition had to generate sufficient disease volume to ensure stable weekly time series at the city level.

Based on preliminary analyses and previous work exploring the spatial dynamics of the 2009 influenza pandemic [11], we elected to use an ILI definition that includes a direct mention of influenza, or fever combined with a respiratory symptom, or febrile viral illness (ICD-9 487-488 OR [780.6 and (462 or 786.2)] OR 079.99). Code 079.99 was identified as the most commonly used diagnosis code for patients for whom the physician prescribed oseltamivir during the pandemic period. Few patients received an influenza specific code 487–488, a finding that may reflect that few physician offices utilized rapid influenza tests during the pandemic, following CDC guidelines to focus laboratory resources on the most severe cases [16]. To investigate the specificity of IMS-ILI data for influenza and test the suitability of IMS data for monitoring other winter-seasonal viruses, we also created RSV diagnoses time series (IMS-RSV), based on three RSV-specific ICD-9 codes: 079.6 (RSV infection), 466.11 (RSV-bonchiolitis) and 480.1 (RSV pneumonia).

Weekly incidence time series were compiled and broken down by 10 administrative regions (Text S1) and 4 age groups (under 5 yrs, 5–29, 30–59, 60 and over). Regional population size estimates were available from the US census [17]. To test the performances of the IMS-ILI data locally, we also compiled weekly incidence time series for 21 cities within New York State based on the first 3-digits of the physician's zip code.

All patient records and information were anonymized and de-identified; all records were part of routinely collected information for health insurance purposes. In keeping with similar epidemiological analyses of large-scale insurance administrative databases [11], [12], [13], [14], no institutional board review was sought. Further, all statistical analyses were based on aggregated incidence time series rather than individual patient-level information.

#### Reference influenza surveillance data

Publicly-available influenza surveillance data from 2003–2010 were obtained from two separate reference systems maintained by the CDC: (1) The Outpatient Influenza-like Illness (ILI) Surveillance Network and (2) the US Influenza Virologic Surveillance System [2] (see also [18]). The CDC-ILI Surveillance system consists of a network of healthcare providers who record the weekly proportion of patients presenting with non-specific signs and symptoms that meet a case definition of influenza like illness [1]. CDC Virus Surveillance data come from ∼140 laboratories throughout the US that report the total number of respiratory specimens tested and the number of laboratory tests positive for influenza virus on a weekly timescale [1]. Both of these databases are available at the national and regional levels (Text S1).

#### Negative control reference surveillance data (RSV)

We also compiled weekly national data on laboratory-confirmed RSV activity during 2003–2010 from the CDC's National Respiratory and Enteric Virus Surveillance System [9]. These data were used both to validate the IMS-RSV indicator and as a non-influenza control for IMS-ILI indicators. If IMS-ILI data are specific of influenza activity, we would expect IMS-ILI time series to be strongly correlated with reference influenza surveillance time series, and far less so with reference RSV surveillance time series.

#### Local influenza surveillance data

To evaluate the performances of IMS-Health at a local level, we focused on New York City, where disease surveillance is particularly well-established [3], [19], [20]. We used weekly city-level syndromic ILI surveillance during 2003–2010, based on 95% of emergency department visits, which are reference influenza time series included in the CDC-ILI dataset for the broader mid-Atlantic region [3], [19], [20].

We also document 2009 pandemic disease patterns in 21 cities or county regions of New York State based on medical claims data, as there was important spatial heterogeneity in pandemic activity in this state [3], [11].

### Statistical approach

#### Study period and spatial scales

We compared weekly ILI and RSV indicators based on medical claims with weekly reference surveillance data from July 2003 to June 2010. This period included 6 pre-pandemic seasons (July 2003–June 2004, July 2004–June 2005, July 2005–June 2006, July 2006–June 2007, July 2007–June 2008, July 2008–April 2009), and the spring and fall 2009 A/H1N1 pandemic waves (May–Aug 2009 and September 2009–June 2010).

The spatial scale of most of our analyses was the region or city, except for comparisons with RSV laboratory-confirmed surveillance, for which retrospective data were available only nationally.

#### Influenza incidence measures

For week *t* and region *i*, we defined the IMS-ILI incidence indicator as the ratio of all ILI visits in the IMS dataset to the total number of IMS visits that week, per 100,000 population, as in [11]:

IMS_ILI_incidence(t,i)  = [(IMS_ILI_t,i_/IMS_visits_t,i_)]*[(population_i_/100,000)].

This indicator is an extension of the ILI incidence ratio used by CDC and New York City [18], with additional standardization for population size. The IMS-RSV incidence indicator was created in the same way as the IMS-ILI indicator. To aggregate IMS data nationally, we weighted weekly regional incidence estimates by the number of physicians participating in surveillance in each week and region.

We defined laboratory-confirmed influenza virus activity in region *i* and week *t* as the standardized number of influenza specimens testing positive for influenza, following:

Virus_activity(t,i) = flu_positives_t,i,_/total_specimens_tested_s,i_

Where flu_positives_t,i,_ is the number of samples testing positive for influenza in week *t* and region *i*, total_specimens_tested_s,i_ is the total number of samples tested in influenza season *s* and region *i* [7]. An alternative is to standardize by the weekly number of specimens tested (weekly percent virus positive), but this indicator is more sensitive to sampling issues at the regional level, especially at the start and end of the influenza season. We used the same standardization for RSV laboratory-surveillance data.

#### Weekly correlation between surveillance time series

To investigate whether the IMS-ILI indicator provided accurate measurement of influenza epidemic patterns and following earlier work [3], [18], we computed the week-by-week Pearson's correlation between IMS-ILI and reference influenza surveillance time series Since the estimated correlation could be explained in part by shared winter seasonality across disease datasets, we also computed the expected level of correlation under the null hypothesis where correlation originates exclusively from winter seasonality rather than influenza-specific factors. To do so, we generated 1,000 simulated datasets for each region and surveillance system by permuting seasons.

A complementary test of the specificity of medical claims for influenza surveillance was obtained by computing the correlation between the IMS-ILI indicators and reference RSV surveillance time series. These indicators share common winter seasonality but are presumably prone to independent yearly and weekly fluctuations specific to influenza and RSV.

#### Influenza and RSV peak timing

We compared the peak timing of disease activity each season (defined as the week of maximum weekly IMS- ILI incidence, IMS-RSV incidence, CDC-ILI incidence, CDC influenza virus activity, and CDC RSV activity in any given season). We computed the difference in peak timing per season, and report the average and range of differences by region.

#### Seasonal intensity of influenza and RSV epidemics

To obtain a summary measure of influenza intensity by season, we applied Serfling seasonal regression model to both medical claims and reference ILI time series [3], [21], [22], [23]. The Serfling approach assumes that background non-influenza ILI incidence follows a seasonal pattern, and that background seasonality does not fluctuate between years. In this approach, a linear regression model including harmonic terms and time trends is fitted to non-influenza weeks (May-Oct), after exclusion of both pandemic seasons. The model provides a seasonal baseline of the expected level of ILI activity when influenza does not circulate. In consequence, the burden of influenza on ILI can be estimated as the cumulative difference between observed and baseline ILI each respiratory season, which is a proxy for seasonal influenza intensity. We repeated the analysis for all age and age-specific data. A similar approach was used to compute seasonal estimates of RSV intensity from weekly IMS-RSV indicators.

From laboratory-confirmed influenza time series, we defined influenza seasonal intensity as the total virus percent positive each respiratory season ( = sum of all influenza positive specimens/sum of all specimens tested during the season), as in CDC summary reports [2]. A similar approach was used to compute RSV intensity from weekly laboratory-confirmed RSV surveillance. No age breakdown was available for CDC's viral activity data.

All analyses where performed in R; scripts are available from the authors upon request.

## Results

### Regional comparisons

#### Overall patterns in influenza incidence

**Figure 1:**
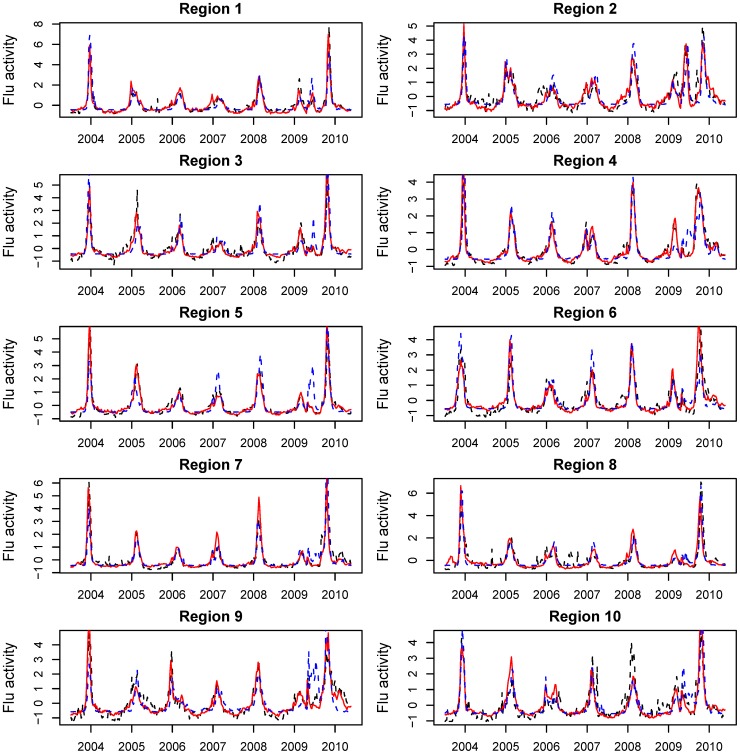
Weekly time series of IMS Heath medical claims influenza-like-illness (ILI, red line), and reference influenza surveillance time series, including CDC-ILI (dashed black line), and CDC laboratory influenza virus surveillance (dashed blue line), by region, from July 7, 2003 through May 23, 2010. All time series have been rescaled for graphing purposes.

Weekly regional influenza time series are displayed in Figure 1 for three surveillance systems for the period 2003–2010: IMS-ILI, CDC-ILI and CDC laboratory-confirmed influenza viral activity. All datasets were characterized by strong winter seasonal peaks during November-March, except for the unusual occurrence of spring and fall pandemic peaks in 2009 in all regions. Between-season fluctuations in influenza intensity were also observed, as expected from variation in circulating strains and levels of population immunity. All three surveillance systems captured the moderately-sized spring 2009 pandemic wave in New England, and a large spring wave in the New York City metropolitan region. In other regions, laboratory-confirmed virus activity tended to overestimate the impact of the pandemic spring wave, relative to the other systems (Figure 1).

#### Week-to-week influenza incidence correlation

All three influenza surveillance datasets were strongly synchronous, as evidenced by high average week-to-week correlation across the 10 regions (Table 1, all P<0.001). The IMS-ILI dataset was particularly highly correlated with the two CDC surveillance systems, with an average correlation above 0.89 (range across regions and indicators, 0.80–0.97, P<0.001). The lag maximizing the correlation between IMS-ILI and CDC-ILI time series ranged between 0 and 1 wk across regions, indicating a slight lead in IMS Health data. Similarly, IMS-ILI led CDC viral activity indicators by 0–1 wk.

**Table 1:** Correlation in timing, weekly incidence and seasonal intensity of influenza epidemics, as measured by 3 surveillance systems: IMS-ILI, CDC-ILI, and CDC laboratory surveillance, by region from 2003–04 to 2009–10.

Correlation outcome	Region 1 (Boston)	Region 2 (New York City)	Region 3 (Wash. DC)	Region 4 (Atlanta)	Region 5 (Chicago)	Region 6 (Dallas)	Region 7 (Kansas City)	Region 8 (Denver)	Region 9 (San Francis.)	Region 10 (Seattle)	Avg. (95% CI)
*IMS-ILI v. CDC-ILI*											
Wkly inc. (lag)^a^	**0.94 (1)**	**0.88 (0)**	**0.90 (1)**	**0.93 (1)**	**0.97 (1)**	**0.90 (1)**	**0.94 (1)**	**0.90 (1)**	**0.90 (1)**	**0.83 (0)**	**0.91 (0.88; 0.93)**
Peak week	**0.93**	**0.95**	**0.87**	**0.94**	**0.98**	**0.95**	**0.99**	0.70	**0.99**	**0.80**	**0.91 (0.85; 0.97)**
Seas. intensity	**0.92**	**0.63**	0.24	0.62	0.62	0.09	**0.76**	0.19	0.54	0.54	0.52 (0.33; 0.68)
*IMS-ILI v. CDC viral surveillance*											
Wkly inc. (lag)^b^	**0.89 (1)**	**0.87 (1)**	**0.89 (1)**	**0.93 (1)**	**0.85 (0)**	**0.90 (1)**	**0.88 (0)**	**0.93 (1)**	**0.80 (1)**	**0.94 (1)**	**0.89 (0.86; 0.91)**
Peak week	**0.93**	**0.97**	**0.97**	**0.92**	**0.94**	**0.99**	**0.99**	**0.98**	**0.99**	**0.97**	**0.97 (0.95; 0.99)**
Seas. intensity	0.06	0.15	0.05	−0.02	−0.27	0.66	0.23	0.34	−0.18	0.10	0.11 (−0.05; 0.28)
*CDC-ILI v. CDC viral surveillance*											
Wkly inc. (lag)^c^	**0.91 (0)**	**0.83 (0)**	**0.83 (0)**	**0.92 (0)**	**0.85 (0)**	**0.86 (0)**	**0.89 (0)**	**0.90 (0)**	**0.71 (1)**	**0.82 (1)**	**0.85 (0.81; 0.89)**
Peak week	**0.98**	**0.86**	**0.94**	**0.96**	**0.96**	**0.95**	**0.97**	**0.76**	**0.99**	**0.79**	**0.92 (0.87; 0.97)**
Seas. intensity	0.35	0.54	0.62	0.58	−0.02	0.33	0.75	0.81	0.38	0.52	0.49 (0.34; 0.63)

Values indicate Pearson correlation coefficients; values in bold are significant. For ILI time series, seasonal intensity is based on excess incidence over baseline each season, estimated from Serfling seasonal regression. For CDC laboratory surveillance, seasonal intensity is based on the cumulative percent virus positive each season (sum of influenza virus positives/sum of respiratory specimens tested).

a Lag maximizing the correlation between the two indicators indicated in parentheses. A positive lag indicates that IMS-ILI surveillance is ahead of CDC-ILI.

b A positive lag indicates that IMS-ILI is ahead of CDC viral surveillance.

c A positive lag indicates that CDC-ILI surveillance is ahead of CDC laboratory-confirmed viral activity.

A season-by-season analysis revealed a clear drop in correlation during the 2009 spring pandemic period, relative to pre-pandemic seasons (average correlation in spring 2009, 0.32–0.45, range across regions (−0.50; 0.89); Text S1). All correlations returned to high levels during the fall 2009 pandemic period (rho>0.88).

Permutations of respiratory seasons resulted in much lower week-to-week correlation between the regional incidence time series than in the original analysis (correlation ranging between −0.08; 0.67 across shuffled datasets; P<0.001 for difference with original data). These results indicate that winter seasonality alone was insufficient to explain the high level of synchrony observed between these influenza surveillance time series.

#### Comparison of influenza peak timing

**Figure 2:**
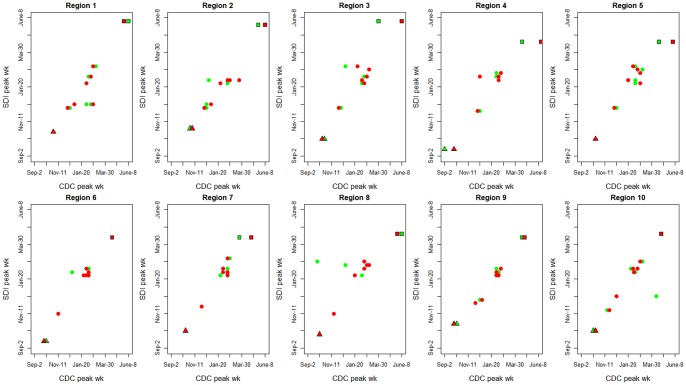
Synchrony in peak timing between the 3 surveillance systems, IMS-ILI, CDC-ILI, and CDC laboratory-confirmed virus activity, by region. Red symbols illustrate the comparison between IMS-ILI (y-axis) and CDC laboratory surveillance (x-axis); while green symbols represent the comparison between IMS-ILI (y-axis) and CDC-ILI (x-axis). Dots represents 6 pre-pandemic seasons, 2003–04 to 2008–09; squares represent the spring 2009 pandemic wave, and triangles the main pandemic wave in fall 2009.

Peak timing provides a complementary measure of synchrony between influenza surveillance systems. All three systems exhibited high agreement in estimated influenza peak week across all regions (average correlation ≥0.91, Table 1). Synchrony was particularly high between IMS-ILI and CDC laboratory-confirmed virus activity (average rho = 0.97), including during the pandemic period (Table 1, Figure 2). Results were robust to national aggregation of the data (Figure S1).

#### Comparison of seasonal influenza intensity

**Figure 3:**
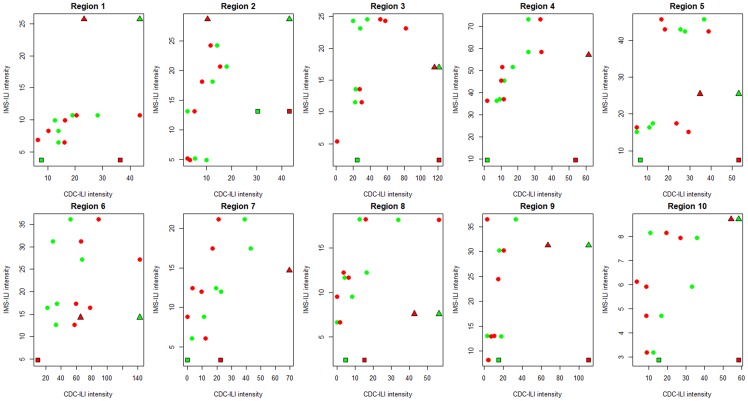
Comparison of influenza seasonal intensity measured by the 3 surveillance systems, IMS-ILI, CDC-ILI, and CDC laboratory-confirmed virus activity, by region. Red symbols illustrate the comparison between IMS-ILI (y-axis) and CDC laboratory surveillance (x-axis); while green symbols represent the comparison between IMS-ILI (y-axis) and CDC-ILI (x-axis). Dots represents 6 pre-pandemic seasons, 2003–04 to 2008–09; squares represent the spring 2009 pandemic wave, and triangles the main pandemic wave in fall 2009. As regards ILI, intensity is based on excess incidence over baseline each season, estimated from Serfling seasonal regression. As regards CDC virus surveillance, intensity is based on the cumulative percent positive each season (sum of virus positives/sum of specimens tested).

Although the above comparisons of week-to-week fluctuations and peak timing suggest a high level of synchrony between surveillance datasets, especially in inter-pandemic seasons, there was less agreement in seasonal estimates of “excess” influenza intensity derived from Serfling models (Table 1, Figure 3). Correlation between seasonal excess ILI estimates in IMS and CDC surveillance was moderate (average rho = 0.52, range across regions 0.09–0.92), with non-significant correlations in 7 of 10 regions (P≥0.29). Correlation in seasonal intensity was even weaker between IMS surveillance and CDC virus activity (average rho = 0.11), with no region exhibiting significant correlation.

Similarly, correlation in seasonal influenza intensity estimates was low between the two surveillance systems maintained by CDC (average rho = 0.49 between CDC-ILI and CDC virus activity), with only 2 regions showing significant correlation (Table 1).

#### Age patterns

Next, we repeated the previous analyses stratified by four age groups (Table 2; see also Figure S2 for age-specific time series). Correlations in weekly incidences and peak timing remained excellent between IMS-ILI and CDC-ILI for intermediate age groups (school-age children and young adults, average rho≥0.84), and more moderate among younger children and seniors. In contrast, agreement in seasonal intensity estimates between surveillance systems was strongest among seniors. Age-specific CDC-ILI time series tended to be noisier for seniors, as compared with other age groups, with most intense fluctuations in the first 3 years of the study period. IMS-ILI time series in children under 5 years displayed semi-annual peaks in summer and winter in North-Eastern US, which remained unexplained and did not coincide with influenza or RSV activity (Figure S3). Nevertheless, pediatric IMS surveillance time series accurately captured non-overlapping influenza and RSV activity in South-Eastern US (Figure S4).

**Table 2:** Age-specific correlations between IMS-ILI and CDC-ILI on a regional scale, 2003–04 to 2009–10.

Outcome/Age group	<5 yrs	5–19 yrs	20–64 yrs	Over 65 yrs
Weekly incidence	**0.72 (0.64–0.81)**	**0.89 (0.81–0.95)**	**0.84 (0.72–0.93)**	**0.70 (0.41–0.86)**
Peak week	**0.76** (0.41**–0.96**)	**0.98 (0.92–1)**	**0.87** (0.5**–0.99**)	0.68 (0.21**–0.96**)
Intensity	0.54 (0.27**–0.80**)	0.28 (−0.33**–0.93**)	0.64 (0.43**–0.9**)	**0.76** (0.53**–0.97**)

Values indicate average Pearson correlation coefficients across 10 regions (range is provided in parentheses); values in bold are significant (P<0.05).

### Analysis of RSV patterns

To check the specificity of medical claims data to monitor influenza activity, we compared IMS-ILI indicators against national reference surveillance data for RSV. The IMS-ILI indicator was significantly less correlated with reference RSV data than with reference influenza indicators on a national scale (week-to-week correlation, rho≥0.93 with CDC-ILI and influenza viral activity, v. rho = 0.33 with laboratory-confirmed RSV, *P*<0.05, Table 3). This analysis also confirmed the slight lead of the IMS data over traditional surveillance when datasets are aggregated nationally. Similarly, peak timing was more synchronous between IMS-ILI and reference influenza surveillance than with reference RSV data. Seasonal estimates of epidemic intensity were significantly correlated between the IMS-ILI and CDC-ILI surveillance systems, but not in the other datasets (Table 3).

**Table 3:** Correlations between IMS indicators for ILI and RSV activity vs traditional influenza surveillance datasets maintained by the CDC.

	IMS-ILI indicator and:			IMS RSV indicator and CDC RSV activity
Outcome	CDC-ILI	CDC influenza viral activity	CDC RSV activity	
Weekly incidence	**0.97** (1)	**0.93** (0)	**0.33** (0)*	**0.94** (0)
Peak week	**0.99**	**0.97**	0.21	**0.97**
Intensity	**0.90**	−0.10	0.43	**0.77**

Correlation between IMS-ILI and CDC RSV laboratory-confirmed viral activity can be considered as a control comparison testing the specificity of IMS-ILI for influenza surveillance. Analyses performed at the national scale, 2003–04 to 2009–10. Values indicate Pearson correlation coefficients; values in bold are significant (P<0.05).

*Correlation for this control comparison is significantly weaker than for the other comparisons (P≤ 0.05), indicating that the IMS-ILI indicator is specific of weekly influenza activity while the IMS RSV indicator is specific of weekly RSV activity.

We also check the consistency of RSV indicators derived from medical claims data with reference RSV time series. The IMS-RSV indicator was highly synchronized with reference RSV laboratory surveillance data, with a 0-week lag maximizing correlation between these datasets (rho≥0.94, *P*<0.0001, for week-to-week incidence and peak week correlation, Table 3). Seasonal estimates of RSV intensity were moderately correlated between the two datasets (rho = 0.77, *P* = 0.03, Table 3).

### City-level influenza disease patterns

**Figure 4:**
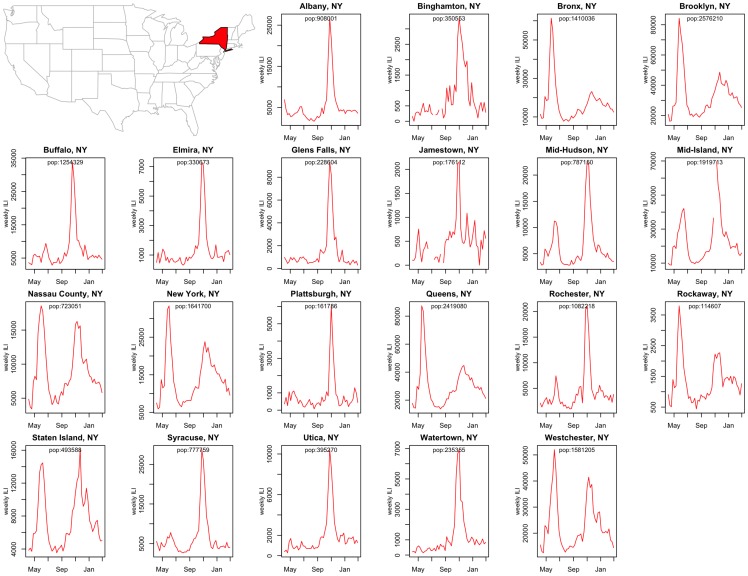
Spatial variation in local influenza activity: 2009 influenza pandemic patterns in 21 cities and county regions of New York State. Weekly IMS-ILI indicators are represented for the period May 2009 to April 2010.

Next, we explored city-level disease curves (Figure 4). We focus on the 2009 pandemic period in 21 cities and counties in New York State, for which spatial heterogeneity has been well documented [3], [11]. Influenza pandemic patterns appear highly heterogeneous at such a local scale. In particular, the New York City boroughs display intense influenza activity in spring 2009 followed by a moderate outbreak in fall 2009, while locations in upstate New York experienced a dominant fall wave. Comparison of IMS-ILI time series for 4 boroughs of New York City with available data (Manhattan, Queens, Bronx and Brooklyn), revealed high consistency in IMS surveillance within this local area (Figure 4: pairwise weekly correlation ≥0.87; P<0.0001). This analysis confirms the robustness of the IMS system for local disease monitoring, as we would expect high population connectivity within New York City, resulting in highly synchronous disease patterns between boroughs.

**Figure 5:**
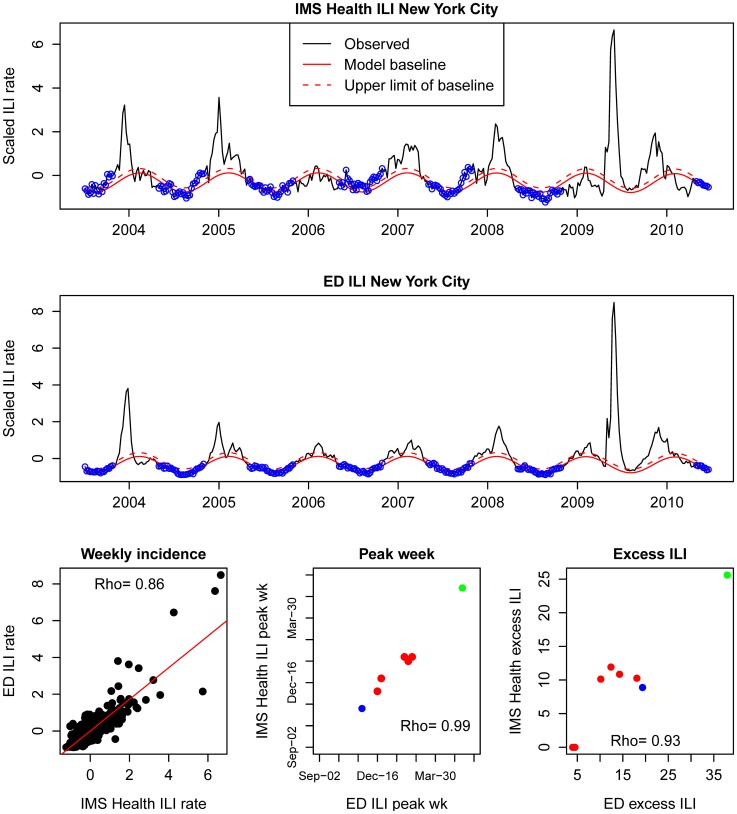
Comparison of the IMS-ILI indicator for New York City (top panel) against reference surveillance data available locally from emergency department (ED) visits (middle panel). Seasonal regression models are fitted to both time series, as explained in the text (blue dots represent non-epidemic observations used to fit the model). The bottom panels display correlations in weekly incidences, peak weeks and seasonal intensity estimates (red dots; seasonal influenza; blue dots; fall 2009 pandemic wave; green dot; spring 2009 pandemic wave) between the IMS dataset and reference data.

Additional comparison of IMS-ILI indicators for New York City against locally available reference influenza surveillance data reveals strong synchrony between datasets (Figure 5). There was high correlation in weekly incidences (rho = 0.86, P<0.0001), and excellent correlation in peak timing (rho = 0.99, P<0.0001), and seasonal intensity (rho = 0.93, P<0.001). Reassuringly, the spring wave of the 2009 pandemic appeared as an outlier in both datasets, confirming the unusually pronounced first wave of A/H1N1 pandemic virus activity in this city.

## Discussion

Although there has been important progress in influenza surveillance systems in recent years [1], [3], [19], [20], highly-resolved spatial disease data based on medical diagnoses are still lacking [3]. We have shown here that medical claims-based ILI indicators derived from IMS Health accurately capture weekly fluctuations and timing of influenza activity in all 10 US regions. Our analyses indicate that high-volume all-age ILI indicators are reliable during inter-pandemic and pandemic seasons and can be broken down by age groups and fine geographical areas. Further, comparisons against control RSV surveillance time series confirm the specificity of the IMS-ILI indicator for influenza. In contrast, we found weak agreement between the various surveillance systems in their seasonal estimates of influenza intensity, especially for school-age children.

The level of correlation found between reference CDC-ILI surveillance data and IMS-ILI indicators (range across regions 0.83–0.97) compares favorably to that reported for Google Flu Trends (range across regions 0.84–0.96, [18]). Google Flu Trends were deliberately calibrated against CDC-ILI surveillance, relying on a set of Google search queries that optimized the correlation between the two systems [24]. However, Google Flu Trends were unable to capture the spring wave of the 2009 pandemic, prompting a major revision of the algorithm [3], [25]. In contrast, IMS-ILI surveillance was not calibrated against CDC surveillance but nevertheless accurately captured increased influenza activity in the Boston and New York regions in May-June 2009, two regions particularly affected by the spring pandemic wave [3], [15]. Other limitations of the updated Google Flu Trends algorithm include the failure to capture the correct timing and intensity of the recent and well-publicized 2012–13 influenza season at various geographic scales, perhaps due to changes in media attention [3], [4]. Importantly, the gold standard for influenza surveillance remains laboratory-confirmed viral activity. It is noteworthy that influenza viral activity was more highly correlated with IMS-ILI surveillance than with Google Flu Trends (rho = 0.89 v. 0.70 for regional data, this study and [18]). These encouraging results suggest that IMS-ILI may be a more specific indicator of influenza patterns than Google Flu Trends.

Using retrospective IMS Health data based on the actual day of the physician's visit, we identified a lead of about 0–1 week between IMS-ILI and reference CDC influenza indicators, indicating increased timeliness of IMS data. The slight lag associated with reference surveillance systems, relative to IMS Health data, may result from delays associated with laboratory testing and temporal aggregation of disease encounters. In contrast, past work suggests there is essentially no lag between reference influenza surveillance systems and Google Flu Trends in the US [18]. Here we focused on providing a historical validation of the IMS Health data, and did not attempt to test the real-time availability of these data. However, if IMS Health claims data were to be used for prospective surveillance purposes, timeliness would depend on the ability to generate timely weekly reports, perhaps based on a known pattern of trickling in of medical claims following physician encounters. Our strategy of stabilizing the IMS-ILI indicator by denominating with the total number of visits will likely secure the reliability of IMS Health data in near real-time use, even before all claims have been submitted.

The level of agreement in estimates of seasonal intensity by the various influenza surveillance systems was only moderate or weak, especially for school-age children. This is not surprising perhaps as the intensity of an epidemic is more difficult to capture than its timing [26]. As with any ILI surveillance system, IMS-ILI and CDC-ILI data include a background level of activity originating from the contribution of non-influenza respiratory pathogens, which can be filtered by seasonal regression models [22]. This approach however was insufficient to provide a high level of correlation in seasonal intensity estimates at the regional scale in our study. It is possible that different proportions of pediatricians in the IMS and CDC surveillance systems, sample sizes issues, and/or the increased contribution of non-influenza pathogens at younger ages, obscures comparisons of intensity. Interestingly, IMS-ILI data in young children were markedly different from reference RSV time series, suggesting these indicators do not capture RSV activity patterns in a major way.

Although laboratory-confirmed virus activity is highly specific for influenza, it remains unclear whether such data should be considered a gold standard for epidemic intensity. Between- season variation in sampling intensity and diagnostic sensitivity may bias the percent virus positive metric, as can fluctuations in co-circulating respiratory pathogens. For instance, the CDC-ILI and IMS-ILI indicators had similar intensity in spring 2009, while at the same time departing from influenza virus surveillance data, suggesting an inflated impact of the spring 2009 pandemic wave in laboratory surveillance. Intensified sampling of respiratory specimens during the first few weeks of pandemic activity, combined with a minimal contribution of co-circulating respiratory pathogens in the spring, would explain these discrepancies. On the other hand, the percent virus positive metric never exceeds ∼40% even in the most severe influenza seasons, perhaps due to detection issues [27]. Consequently, others have proposed a combination of ILI and percent virus positive as the most appropriate indicator of influenza intensity [27]. Although attractive and particularly well correlated with influenza-related mortality [28], this composite indicator is not available at a local scale where viral sampling remains too sparse. Similarly, the integration of local Google Flu Trends indicators with regionally-available percent virus positive data has been put forward to monitor influenza activity in US cities [29]. The performances of this hybrid surveillance approach should be quantified however, especially as Google Flu Trends is prone to important under- and over-estimation issues [3]. Overall, further theoretical and simulation work should concentrate on identifying the most appropriate indicators of disease intensity at weekly and seasonal time scales and evaluate putative biases.

Although we were unable to systematically validate city-level IMS-ILI indicators throughout the US due to unavailability of a gold standard at the relevant spatial scale, our comparison focused on New York City was promising. At the local level, IMS-ILI data revealed important spatial heterogeneity in pandemic patterns between cities in New York State, together with great consistency between well-connected boroughs of New York City, indicating the robustness of this system to monitor local disease spread.

Although a thorough validation of IMS-RSV disease indicators at local and regional scales was beyond the scope of this study, we noted a clear promise in the IMS data for tracking RSV activity. More work in this area would be worthwhile as local indicators of RSV activity are urgently needed to guide the timing of prophylaxis in individual locations [9], [10], until vaccines become available. Further, availability of local RSV data could help shed light on the transmission dynamics of this less-studied pathogen and the surprising level of spatial heterogeneity in seasonal epidemics [10], [30].

Our study is subject to several limitations. Our study period was relatively short, 2003–2010, which includes only 6 inter-pandemic and 2 pandemic seasons. Although IMS surveillance started in 2001, the number of participating physicians and data volume increased substantially in the first two years, which we chose to discard from this study. Nevertheless, we were able to capture a variety of influenza seasons dominated by all 3 influenza subtypes, ranging from mild and double-peaked winter epidemics (2006–07), to the localized spring 2009 pandemic wave, and the very spiky and unusual fall 2009 pandemic wave. Further, we did not test the performances of IMS Health data for real time surveillance in a prospective manner, which would require a careful study of the dynamics of accumulation of claims into the IMS Health data warehouse.

In summary, we have shown that the medical claims-based surveillance is a very promising tool to study influenza and RSV activity at regional and local scales in inter-pandemic and pandemic seasons. While there has been great progress in the last decade in building sophisticated spatial simulation models of pandemic influenza spread in the US and globally [31], [32], [33], proper model validation against empirical disease patterns is still sparse due to the lack of fine-grained epidemiological data [11]. There has been considerable interest in novel surveillance systems such as Google Flu Trends to fill the void, particularly in large cities such as New York [34]. It has recently become clear however that search query indicators may not always capture true disease patterns and miss critical epidemiological features such as out-of-season pandemic outbreaks [3]. Alternative choices include high-volume medical databases maintained in the private sector, such as IMS Health, and similar data streams generated in the public sector, such as Biosense [35], [36] and Electronic Health Records from ambulatory clinics [37]. One important obstacle to a wider use of large medical claims databases is perhaps their prohibitive cost for public health research, although this will become less of an issue in the future as the cost of electronic health data is declining. In conclusion, we believe medical claims data offer a unique opportunity to provide rapid disease information to the scientific and public health communities for local situational awareness, to refine existing influenza transmission models, and support pandemic response in future outbreaks.

## Supporting Information

Figure S1 **Synchrony in peak timing between IMS-ILI indicators and reference surveillance systems, including CDC-ILI (A), CDC laboratory-confirmed influenza virus activity (B), and CDC laboratory-confirmed RSV activity (C).** Comparisons are based on nationally-aggregated data. Red circles represent 6 pre-pandemic seasons, 2003–04 to 2008–09; green dots represent the spring 2009 pandemic wave and blue dots the main pandemic wave in fall 2009.
(TIF)

Figure S2 **Age-specific ILI time series in the Boston region (region 1) based on CDC surveillance and IMS Health.**
(TIF)

Figure S3 **Comparison of IMS-ILI, CDC-ILI and IMS-RSV indicators in the Boston region (region 1) for children under 5 yrs.** Note the semi-annual pattern of activity in IMS-ILI data (large peak in winters and smaller peaks in summers), which is most pronounced in the North-East. The bottom panel suggests that IMS-ILI does not align with RSV activity but instead accurately captures unusually early influenza activity in fall 2003 (severe A/H3N2 Fujian season) and fall 2009 (A/H1N1 pandemic; see also Figure S4).
(TIF)

Figure S4 **Comparison of IMS-ILI, CDC-ILI and IMS-RSV indicators in the Atlanta region (region 4), for children under 5 yrs.** In this region, RSV is known to display very early timing of activity and predates influenza in most years, except for the 2003–04 influenza season and 2009 fall pandemic wave. The bottom panel confirms that the pediatric IMS-ILI indicator does not capture RSV activity patterns (see also Figure S3).
(TIF)

Text S1 **Description of supplementary text.**
(DOC)
