# Supplementary figures and images for: Demonstrating the Use of High-Volume Electronic Medical Claims Data to Monitor Local and Regional Influenza Activity in the US
Source: PLoS One. 2014 Jul 29;9(7):e102429. doi: 10.1371/journal.pone.0102429 (PMC4114744; doi:10.1371/journal.pone.0102429)

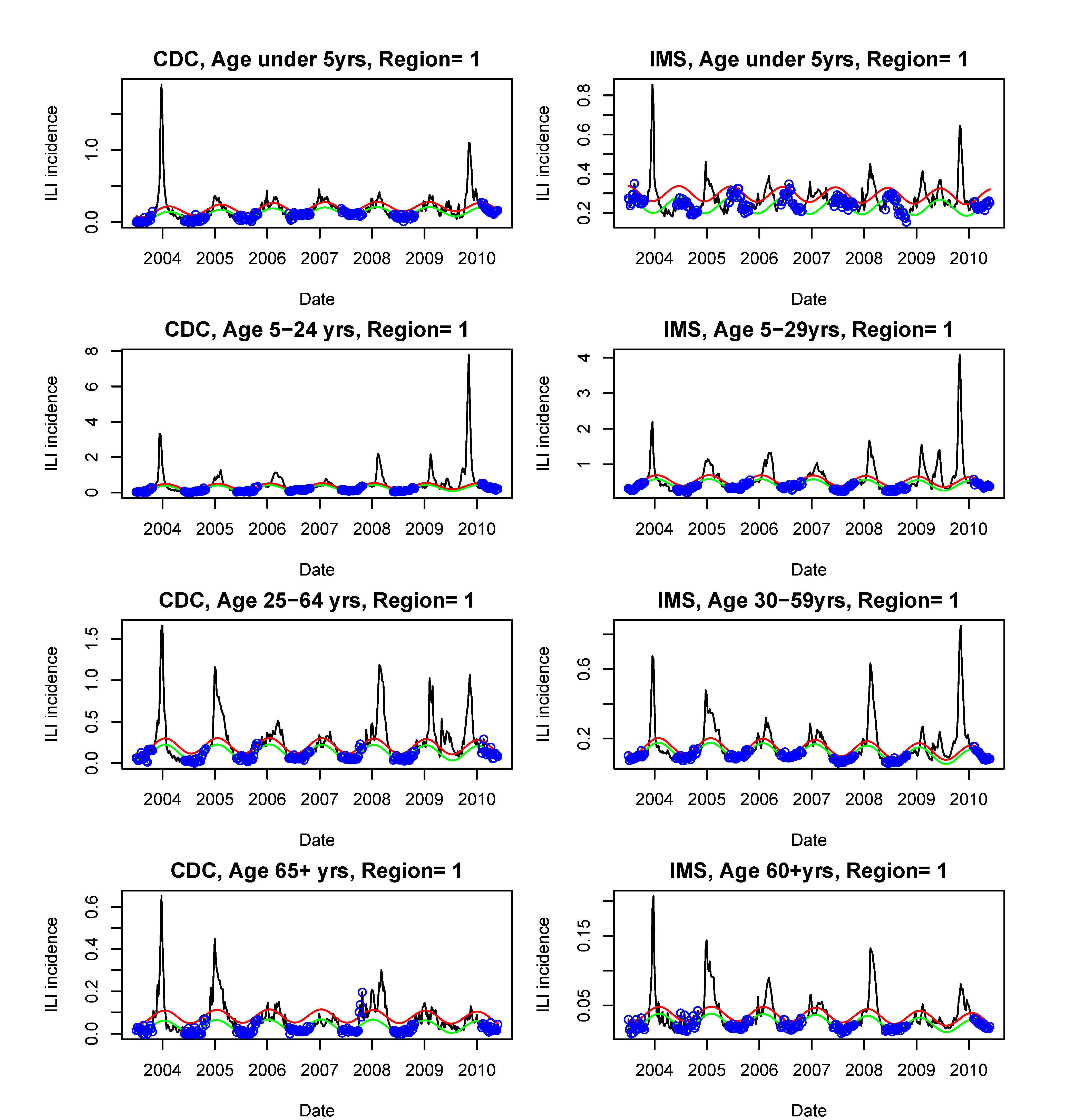

Supplement: Figure S2 — Age-specific ILI time series in the Boston region (region 1) based on CDC surveillance and IMS Health. (TIF) [file pone.0102429.s002.tif]
