# Supplementary material for: Demonstrating the Use of High-Volume Electronic Medical Claims Data to Monitor Local and Regional Influenza Activity in the US
Source: PLoS One. 2014 Jul 29;9(7):e102429. doi: 10.1371/journal.pone.0102429 (PMC4114744; doi:10.1371/journal.pone.0102429)
Supplement: Text S1 — Description of supplementary text. (DOC) [file pone.0102429.s005.doc]

**Demonstrating the use of high-volume electronic medical claims data to monitor local and regional influenza activity in the US: Supplementary Information**

**Cécile Viboud**^1^**, Vivek Charu**^1,2^**, Donald Olson^3^, Sébastien Ballesteros**^4^**, Julia Gog**^1,5^**, Farid Khan**^6^**, Bryan Grenfell**^1,4^**, Lone Simonsen**^1,7^

^1^ Fogarty International Center, National Institutes of Health, Bethesda, MD, USA.

^2^ Johns Hopkins University, Baltimore, MD, USA

^3^ New York City Department of Health and Mental Hygiene, New York, NY, USA

^4^ Department of Evolutionary Biology, Princeton University, Princeton, NJ, USA.

^5^ Department of Applied Mathematics and Theoretical Physics, University of Cambridge, Cambridge, UK

^6^ IMS Health, Plymouth Meeting, PA, USA.

^7^ Department of Global Health, School of Public Health and Health Services, George Washington University, Washington, DC, USA.

We provide a series of supplementary analyses in support of the correlation estimates detailed in the main text and some underlying epidemiologic information.

**Supplementary Table 1:** Department of Health and Human Services (HHS) definition of 10 US regions.

| **HHS Region:** | **Main city** | **States:** |
| --- | --- | --- |
| Region 1: | Boston | CT, ME, MA, NH, RI, VT |
| Region 2: | New York City | NJ, NY |
| Region 3: | Washington DC | DE, DC, MD, PA, VA, WV |
| Region 4: | Atlanta | AL, FL, GA, KY, MS, NC, SC, TN |
| Region 5: | Chicago | IL, IN, MI, MN, OH, WI |
| Region 6: | Dallas | AR, LA, NM, OK, TX |
| Region 7: | Kansas City | IA, KS, MO, NE |
| Region 8: | Denver | CO, MT, ND, SD, UT, WY |
| Region 9: | San Francisco | AZ, CA, HI, NV |
| Region 10: | Seattle | AK, ID, OR, WA |

**Supplementary Table 2:** Characteristics of influenza epidemics based on IMS-ILI medical claims incidence time series, 2003-2010. Estimates are based on nationally-aggregated data.

|  | 2003-04 | 2004-05 | 2005-06 | 2006-07 | 2007-08 | 2008-09 | Spring 2009 pandemic wave (May-Aug 2009) | Fall 2009 pandemic wave (Sep 2009-June 2010) |
| --- | --- | --- | --- | --- | --- | --- | --- | --- |
| Seasonal intensity* | 265.3 | 232.1 | 149.4 | 142.9 | 283.4 | 121.8 | 58.1 | 258.8 |
| Peak timing | Dec 21, 2003 | Feb 15, 2005 | Feb 26, 2006 | Feb 11, 2007 | Feb 17, 2008 | Feb 22, 2009 | May 15, 2009 | Oct 18, 2009 |

* Cumulative ILI incidence in excess of seasonal baseline during respiratory season, based on Serfling approach. Incidence is normalized by the total number of physician visits and population sizes.

**Supplementary Table 3:** Correlation between IMS ILI incidence proxy and CDC ILI by region and season. Values indicate Pearson correlation in weekly time series. Note the drop in correlation during the spring 2009 pandemic wave.

|  | Region 1 (Boston) | Region 2  (New York City) | Region 3  (Wash. DC) | Region 4  (Atlanta) | Region 5  (Chicago) | Region 6 (Dallas) | Region 7 (Kansas City) | Region 8  (Denver) | Region 9  (San Franc.) | Region 10 (Seattle) | Avg.  (95% CI) |  |
| --- | --- | --- | --- | --- | --- | --- | --- | --- | --- | --- | --- | --- |
| 2003/04 | 0.95 | 0.95 | 0.93 | 0.88 | 0.95 | 0.92 | 0.89 | 0.88 | 0.91 | 0.97 | 0.92 (0.9;0.94) | |
| 2004/05 | 0.93 | 0.94 | 0.94 | 0.97 | 0.97 | 0.90 | 0.96 | 0.87 | 0.92 | 0.90 | 0.93 (0.91;0.95) | |
| 2005/06 | 0.90 | 0.61 | 0.90 | 0.95 | 0.93 | 0.87 | 0.96 | 0.78 | 0.88 | 0.71 | 0.85 (0.78;0.92) | |
| 2006/07 | 0.87 | 0.92 | 0.80 | 0.92 | 0.96 | 0.91 | 0.89 | 0.44 | 0.95 | 0.80 | 0.85 (0.75;0.94) | |
| 2007/08 | 0.97 | 0.94 | 0.91 | 0.96 | 0.99 | 0.94 | 0.96 | 0.89 | 0.97 | 0.91 | 0.94 (0.92;0.96) | |
| 2008/09 | 0.93 | 0.83 | 0.93 | 0.94 | 0.98 | 0.89 | 0.90 | 0.88 | 0.92 | 0.92 | 0.91 (0.89;0.94) | |
| 2009 spring pandemic | 0.63 | 0.86 | -0.50 | 0.55 | 0.51 | 0.50 | 0.45 | -0.25 | 0.58 | 0.41 | 0.37 (0.12;0.63) | |
| 2009 fall pandemic | 0.92 | 0.90 | 0.90 | 0.97 | 0.91 | 0.83 | 0.89 | 0.79 | 0.92 | 0.95 | 0.9 (0.86;0.93) | |

**Supplementary Table 4:** Correlation between IMS ILI incidence proxy and CDC laboratory-confirmed virus activity by region and season. Values indicate Pearson correlation in weekly time series. Note the drop in correlation during the 2009 spring pandemic period.

|  | Region 1 (Boston) | Region 2  (New York City) | Region 3  (Wash. DC) | Region 4  (Atlanta) | Region 5  (Chicago) | Region 6 (Dallas) | Region 7 (Kansas City) | Region 8  (Denver) | Region 9  (San Franc.) | Region 10 (Seattle) | Avg.  (95% CI) |
| --- | --- | --- | --- | --- | --- | --- | --- | --- | --- | --- | --- |
| 2003/04 | 0.94 | 0.91 | 0.95 | 0.86 | 0.97 | 0.98 | 0.97 | 0.96 | 0.97 | 0.97 | 0.95 (0.92;0.97) |
| 2004/05 | 0.91 | 0.93 | 0.85 | 0.96 | 0.82 | 0.92 | 0.96 | 0.98 | 0.84 | 0.93 | 0.91 (0.87;0.95) |
| 2005/06 | 0.94 | 0.83 | 0.90 | 0.95 | 0.96 | 0.91 | 0.91 | 0.87 | 0.76 | 0.88 | 0.89 (0.85;0.93) |
| 2006/07 | 0.66 | 0.69 | 0.76 | 0.90 | 0.90 | 0.95 | 0.81 | 0.97 | 0.82 | 0.86 | 0.83 (0.77;0.9) |
| 2007/08 | 0.90 | 0.88 | 0.85 | 0.98 | 0.90 | 0.98 | 0.96 | 0.86 | 0.90 | 0.95 | 0.92 (0.89;0.95) |
| 2008/09 | 0.89 | 0.88 | 0.89 | 0.95 | 0.97 | 0.95 | 0.94 | 0.97 | 0.85 | 0.95 | 0.92 (0.9;0.95) |
| 2009 spring pandemic | 0.89 | 0.56 | 0.64 | -0.05 | 0.11 | 0.37 | 0.22 | 0.07 | -0.11 | 0.51 | 0.32 (0.12;0.52) |
| 2009 fall pandemic | 0.92 | 0.91 | 0.91 | 0.81 | 0.96 | 0.85 | 0.93 | 0.85 | 0.79 | 0.86 | 0.88 (0.84;0.91) |

**Supplementary Table 5:** Correlation between CDC ILI incidence and CDC laboratory-confirmed virus activity by region and season. Values indicate Pearson correlation in weekly time series. Note the drop in correlation during the 2009 spring pandemic wave.

|  | Region 1 (Boston) | Region 2  (New York City) | Region 3  (Wash. DC) | Region 4  (Atlanta) | Region 5  (Chicago) | Region 6 (Dallas) | Region 7 (Kansas City) | Region 8  (Denver) | Region 9  (San Franc.) | Region 10 (Seattle) | Avg.  (95% CI) |  |
| --- | --- | --- | --- | --- | --- | --- | --- | --- | --- | --- | --- | --- |
| 2003/04 | 0.98 | 0.92 | 0.85 | 0.97 | 0.96 | 0.89 | 0.93 | 0.91 | 0.94 | 0.95 | 0.93 (0.9;0.95) | |
| 2004/05 | 0.93 | 0.90 | 0.78 | 0.94 | 0.72 | 0.97 | 0.94 | 0.88 | 0.76 | 0.86 | 0.87 (0.82;0.92) |  |
| 2005/06 | 0.88 | 0.39 | 0.85 | 0.93 | 0.91 | 0.94 | 0.93 | 0.66 | 0.73 | 0.72 | 0.79 (0.69;0.9) |  |
| 2006/07 | 0.86 | 0.65 | 0.48 | 0.98 | 0.88 | 0.89 | 0.89 | 0.47 | 0.80 | 0.79 | 0.77 (0.66;0.88) |  |
| 2007/08 | 0.93 | 0.96 | 0.79 | 0.95 | 0.92 | 0.95 | 0.90 | 0.92 | 0.86 | 0.88 | 0.91 (0.88;0.94) |  |
| 2008/09 | 0.94 | 0.84 | 0.91 | 0.89 | 0.96 | 0.93 | 0.94 | 0.90 | 0.81 | 0.89 | 0.9 (0.87;0.93) |  |
| 2009 spring pandemic | 0.47 | 0.77 | -0.02 | 0.23 | 0.49 | 0.68 | 0.19 | 0.53 | 0.42 | 0.72 | 0.45 (0.29;0.6) |  |
| 2009 fall pandemic | 0.98 | 0.92 | 0.94 | 0.80 | 0.96 | 0.89 | 0.89 | 0.97 | 0.72 | 0.85 | 0.89 (0.84;0.94) |  |
